# Supplementary material for: Antenatal depression and its association with adverse birth outcomes in low and middle-income countries: A systematic review and meta-analysis
Source: PLoS One. 2020 Jan 10;15(1):e0227323. doi: 10.1371/journal.pone.0227323 (PMC6953869; doi:10.1371/journal.pone.0227323)
Supplement: S1 File — (DOCX) [file pone.0227323.s001.docx]

Additional data analysis result of Prenatal Depression and its Effect on Birth Outcomes: A Systematic Review and Meta-analysis of Studies from Low and Middle-income Countries

Abel Fekadu Dadi^12^* Lillian Mwanri^1^ Emma Miller^1^

^1^Flinders University, Health Sciences Building, Sturt Road, Bedford Park, Adelaide, SA, 5001

^2^Institute of Public Health, College of Medicine and Health Sciences, University of Gondar, Gondar, Ethiopia

**Supplementary Appendix**

Contents

[PRISMA checklist 3](#_Toc511218033)

[Search strategy 5](#_Toc511218034)

[NOS quality assessment checklist 9](#_Toc511218035)

[I. Prenatal depression 9](#_Toc511218036)

[II. Prenatal depression and adverse birth outcomes 14](#_Toc511218037)

[Excluded studies and reason for exclusion 16](#_Toc511218038)

[I. Prenatal depression and associated factors 16](#_Toc511218039)

[II. Effect of prenatal depression on birth outcomes 16](#_Toc511218040)

[Pooled prenatal prevalence, forest plot 17](#_Toc511218041)

[Sensitivity analysis for prenatal depression prevalence 18](#_Toc511218042)

[Egger's test for small-study effects 20](#_Toc511218044)

[Funnel Asymmetry for testing publication bias 20](#_Toc511218045)

[Pooled prenatal depression prevalence after trim and fill analysis 22](#_Toc511218046)

[I. Meta-analysis of factors associated with prenatal depression 23](#_Toc511218047)

[1.1. Economic difficulty as a factor for prenatal depression forest plot 23](#_Toc511218048)

[1.2. History of CMD (*depression, anxiety, stressful life event*) as a factor for prenatal depression, forest plot 24](#_Toc511218049)

[1.3. Male preference in the current pregnancy as a factor for prenatal depression, forest plot 25](#_Toc511218050)

[1.4. Bad obstetric history as a factor of prenatal depression, forest plot 26](#_Toc511218051)

[1.5. Poor social support as a risk factor of prenatal depression, forest plot 27](#_Toc511218052)

[1.6. Unfavorable marital condition as a risk factor for prenatal depression, forest plot 28](#_Toc511218053)

[1.7. History of IPV as a risk factor for prenatal depression, forest plot 29](#_Toc511218054)

[Forest plot after trim and fill analysis between prenatal depression and adverse birth outcome 31](#_Toc511218056)

[Reference 32](#_Toc511218057)

# **PRISMA checklist**

| **Section/topic** | **#** | 1. **Checklist item** | **Reported on page #** |
| --- | --- | --- | --- |
| 1. **TITLE** | | |  |
| Title | 1 | 1. Identify the report as a systematic review, meta-analysis, or both. | 1 |
| 1. **ABSTRACT** | | |  |
| Structured summary | 2 | 1. Provide a structured summary including, as applicable: background; objectives; data sources; study eligibility criteria, participants, and interventions; study appraisal and synthesis methods; results; limitations; conclusions and implications of key findings; systematic review registration number. | 2 |
| 1. **INTRODUCTION** | | |  |
| Rationale | 3 | 1. Describe the rationale for the review in the context of what is already known. | 4 |
| Objectives | 4 | 1. Provide an explicit statement of questions being addressed with reference to participants, interventions, comparisons, outcomes, and study design (PICOS). | 4 |
| 1. **METHODS** | | |  |
| Protocol and registration | 5 | 1. Indicate if a review protocol exists, if and where it can be accessed (e.g., Web address), and, if available, provide registration information including registration number. | 6 |
| Eligibility criteria | 6 | 1. Specify study characteristics (e.g., PICOS, length of follow-up) and report characteristics (e.g., years considered, language, publication status) used as criteria for eligibility, giving rationale. | 4 |
| Information sources | 7 | 1. Describe all information sources (e.g., databases with dates of coverage, contact with study authors to identify additional studies) in the search and date last searched. | 4 |
| Search | 8 | 1. Present full electronic search strategy for at least one database, including any limits used, such that it could be repeated. | 5 |
| Study selection | 9 | 1. State the process for selecting studies (i.e., screening, eligibility, included in systematic review, and, if applicable, included in the meta-analysis). | 5 & 9 |
| Data collection process | 10 | 1. Describe method of data extraction from reports (e.g., piloted forms, independently, in duplicate) and any processes for obtaining and confirming data from investigators. | 5 |
| Data items | 11 | 1. List and define all variables for which data were sought (e.g., PICOS, funding sources) and any assumptions and simplifications made. | 5 |
| Risk of bias in individual studies | 12 | 1. Describe methods used for assessing risk of bias of individual studies (including specification of whether this was done at the study or outcome level), and how this information is to be used in any data synthesis. | 6 |
| Summary measures | 13 | 1. State the principal summary measures (e.g., risk ratio, difference in means). | 5 |
| Synthesis of results | 14 | 1. Describe the methods of handling data and combining results of studies, if done, including measures of consistency (e.g., I^2^) for each meta-analysis. | 6 |

| **Section/topic** | **#** | **Checklist item** | **Reported on page #** |
| --- | --- | --- | --- |
| Risk of bias across studies | 15 | Specify any assessment of risk of bias that may affect the cumulative evidence (e.g., publication bias, selective reporting within studies). | 5 |
| Additional analyses | 16 | Describe methods of additional analyses (e.g., sensitivity or subgroup analyses, meta-regression), if done, indicating which were pre-specified. | 6 |
| **RESULTS** | | |  |
| Study selection | 17 | Give numbers of studies screened, assessed for eligibility, and included in the review, with reasons for exclusions at each stage, ideally with a flow diagram. | 9 |
| Study characteristics | 18 | For each study, present characteristics for which data were extracted (e.g., study size, PICOS, follow-up period) and provide the citations. | 10-12 & 17 |
| Risk of bias within studies | 19 | Present data on risk of bias of each study and, if available, any outcome level assessment (see item 12). | Appendix p 7-13 |
| Results of individual studies | 20 | For all outcomes considered (benefits or harms), present, for each study: (a) simple summary data for each intervention group (b) effect estimates and confidence intervals, ideally with a forest plot. | 10-12 & 17 |
| Synthesis of results | 21 | Present results of each meta-analysis done, including confidence intervals and measures of consistency. | 13-14  18-19  Appendix p 15-20 |
| Risk of bias across studies | 22 | Present results of any assessment of risk of bias across studies (see Item 15). | 5  Appendix 9-13 |
| Additional analysis | 23 | Give results of additional analyses, if done (e.g., sensitivity or subgroup analyses, meta-regression [see Item 16]). | 12-13,16,19 |
| **DISCUSSION** | | |  |
| Summary of evidence | 24 | Summarize the main findings including the strength of evidence for each main outcome; consider their relevance to key groups (e.g., healthcare providers, users, and policy makers). | 20-24 |
| Limitations | 25 | Discuss limitations at study and outcome level (e.g., risk of bias), and at review-level (e.g., incomplete retrieval of identified research, reporting bias). | 24 |
| Conclusions | 26 | Provide a general interpretation of the results in the context of other evidence, and implications for future research. | 24 |
| **FUNDING** | | |  |
| Funding | 27 | Describe sources of funding for the systematic review and other support (e.g., supply of data); role of funders for the systematic review. | 24 |

# Literature Search strategy

We searched CINHAL, MEDLINE, Emcare, [PubMed](https://www.ncbi.nlm.nih.gov/pubmed?otool=iauflullib&myncbishare=flinlib), Psych Info [and Scopus](http://ezproxy.flinders.edu.au/login?url=https://www.scopus.com/scopus/home.url) data bases using the following search terms: Pregnant mothers, antenatal mothers, pregnant women, antenatal period, pregnancy, antepartum women, depression, clinical depression, depressed mood, major depressive disorder, depressive symptom, adverse birth outcome, still birth, preterm birth, low birth weight. We included observational studies conducted in Low and Middle income countries, published in between January 2007 and December 2017, and written in English. Search strategy for data bases are given in table 1.

Table 1: Search strategy

| **#** | **Data base** |  |
| --- | --- | --- |
|  | **Search strategy for prenatal depression and factors** | |
|  | [PubMed](https://www.ncbi.nlm.nih.gov/pubmed?otool=iauflullib&myncbishare=flinlib) | Search ((((((Pregnant mothers*) OR (antenatal mothers*) OR (pregnant women*) OR (antenatal period*) OR pregnancy* OR (antepartum women*)) AND ((depression* OR (clinical depression*) OR (depressed mood*) OR(major depressive disorder*) OR (depressive symptom*) OR (psychological morbidity*) OR (major depression*) OR (unipolar depression*)) AND((exposure* OR (risk factor*) OR correlates* OR (associated factors*) OR predictors*) AND (((cross sectional*) OR (crosssectional*) OR survey* OR(case control*) OR (nested case control*)) Sort by: PublicationDate Filters:Publication date from 2007/01/01 to 2017/12/31; Humans; English;MEDLINE; Field: Title/Abstract |
|  | [Scopus](http://ezproxy.flinders.edu.au/login?url=https://www.scopus.com/scopus/home.url) | ( TITLE-ABS-KEY ( pregnan* OR "antenatal mothers" OR "antepartum wom?n" ) ) AND( TITLE-ABS-KEY ( depress* OR "clinical depression" OR "major depressive disorder" OR "depressive symptom" OR "major depression" ) ) AND ( TITLE-ABS-KEY ( exposure* OR"risk factor*" OR correlates* OR "associated factors" OR predictors ) ) AND ( TITLE-ABSKEY( "cross sectional" OR survey* OR "case control" OR "nested case control" ) ) AND( LIMIT-TO ( PUBYEAR , 2017 ) OR LIMIT-TO ( PUBYEAR , 2016 ) OR LIMIT-TO ( PUBYEAR ,2015 ) OR LIMIT-TO ( PUBYEAR , 2014 ) OR LIMIT-TO ( PUBYEAR , 2013 ) OR LIMITTO( PUBYEAR , 2012 ) OR LIMIT-TO ( PUBYEAR , 2011 ) OR LIMIT-TO ( PUBYEAR , 2010 )OR LIMIT-TO ( PUBYEAR , 2009 ) OR LIMIT-TO ( PUBYEAR , 2008 ) OR LIMIT-TO ( PUBYEAR ,2007 ) ) AND ( LIMIT-TO ( DOCTYPE , "ar" ) OR LIMIT-TO ( DOCTYPE , "re" ) OR LIMITTO( DOCTYPE , "sh" ) ) AND ( LIMIT-TO ( SUBJAREA , "MEDI" ) OR LIMIT-TO ( SUBJAREA ,"NURS" ) ) AND ( LIMIT-TO ( EXACTKEYWORD , "Human" ) OR LIMIT-TO ( EXACTKEYWORD ,"Female" ) OR LIMIT-TO ( EXACTKEYWORD , "Pregnancy" ) OR LIMIT-TO ( EXACTKEYWORD ,"Humans" ) OR LIMIT-TO ( EXACTKEYWORD , "Article" ) ) AND ( LIMIT-TO ( LANGUAGE ,"English" ) ) AND ( LIMIT-TO ( SRCTYPE , "j" ) ) |
|  | **Search strategy for adverse birth outcome** | |
|  | [CINAHL](http://ezproxy.flinders.edu.au/login?url=http://search.ebscohost.com/login.aspx?authtype=ip,uid&profile=ehost&defaultdb=cin20)(EBSCO) | Depression during antenatal period)OR (antenatal depression ) OR (depressionduring pregnancy) OR (low mood) OR(feeling sad) OR (depressed mood) OR(depressive symptom) OR (depressivedisorder with peripurum onset) OR (antipartum depressive onset) AND ((adverse birth outcome*) OR (preterm birth) OR (still birth) OR (small birth) OR (small for gestational age) OR (low birth weight) OR (fetal or infant death) OR (congenital anomaly) OR (infant birth outcome) OR (birth defect) OR macrosomia OR (neonatal outcomes) AND (prospective cohort) OR(retrospective cohort) OR (Follow up study) OR(longitudinal study) OR (case control study)OR (nested case control study) AND (exposure* OR (risk factor*) OR correlates* OR (associated factors*) OR predictors* Sort by: PublicationDate Filters:Publication date from 2007/01/01 to 2017/12/31; Humans; English;MEDLINE; Field: Title/Abstract |

# NOS quality assessment checklist

## Prenatal depression

- 1. **Cross sectional studies**

|  | **Selection**  (max-5points) | | | | **Comparability**  (max-two points) | **Assessment of the outcome**  (max-three points) | | **Total Score** |
| --- | --- | --- | --- | --- | --- | --- | --- | --- |
| List of studies | representativeness of the sample | Sample size | Non-respondents | Ascertainment of the risk-factors | The subjects in different outcome groups are comparable, based on the study design or analysis. Confounding factors are controlled. | Ascertainment of the outcome | Stastical test |  |
| Abdelhai R et al 2015 | 1 | 1 | 1 | 1 | 2 | 1 | 1 | 8 |
| Abujilban SA et al 2014 | 1 | 0 | 1 | 1 | 2 | 1 | 1 | 7 |
| Adewuya, A. et al 2007 | 1 | 0 | 1 | 1 | 1 | 1 | 1 | 7 |
| Ajinkya, S., et al 2013 | 1 | 1 | 1 | 1 | 2 | 1 | 1 | 8 |
| Actas, S. et al 2014 | 1 | 1 | 1 | 1 | 2 | 1 | 1 | 8 |
| Alvarado-Esquivel, C., 2016 | 1 | 1 | 1 | 1 | 2 | 1 | 1 | 8 |
| Assefa Gemta, W. 2015 | 1 | 1 | 1 | 1 | 2 | 1 | 1 | 8 |
| Ayele, Tadesse Awoke, 2016 | 1 | 1 | 1 | 1 | 2 | 1 | 1 | 8 |
| Barrios, Y. V. 2015 | 1 | 0 | 1 | 1 | 2 | 1 | 1 | 7 |
| Bavle, A. D. | 1 | 1 | 1 | 1 | 2 | 1 | 1 | 8 |
| Biratu, A. 2015 | 1 | 1 | 1 | 1 | 2 | 1 | 1 | 8 |
| Bisetegn, T. A. 2016 | 1 | 1 | 1 | 1 | 2 | 1 | 1 | 8 |
| Bitew, T et al 1016 | 1 | 1 | 1 | 1 | 2 | 1 | 1 | 8 |
| Bindt C et al 2013 | 1 | 1 | 1 | 1 | 1 | 1 | 1 | 7 |
| Coll CVDN et al 2017 | 1 | 1 | 1 | 1 | 1 | 1 | 1 | 7 |
| de Jesus Silva, Monica Maria, 2016 | 1 | 1 | 1 | 1 | 2 | 1 | 1 | 8 |
| de Moraes, E. V, 2016 | 1 | 1 | 1 | 1 | 2 | 1 | 1 | 8 |
| de Oliveira Mariana, et al 2015 | 1 | 1 | 1 | 1 | 1 | 2 | 1 | 8 |
| Dibaba, Y.2013 | 1 | 1 | 1 | 1 | 1 | 2 | 1 | 8 |
| Dmitrovic, BK et al , 2013 | 1 | 0 | 1 | 1 | 2 | 1 | 1 | 7 |
| Esimai, O., 2008 | 1 | 0 | 1 | 1 | 1 | 2 | 1 | 7 |
| Fadzil, A, 2013 | 1 | 0 | 1 | 1 | 2 | 1 | 1 | 7 |
| Faisal-Cury, A.,2012 | 0 | 1 | 1 | 1 | 1 | 2 | 1 | 7 |
| Gausia, K 2009 | 1 | 1 | 1 | 1 | 2 | 1 | 1 | 8 |
| Gelaye, Bizu, 2017 | 1 | 0 | 1 | 1 | 2 | 1 | 1 | 7 |
| Gemta A et al 2013 | 1 | 1 | 1 | 1 | 1 | 1 | 1 | 7 |
| George, C. 2016 | 1 | 1 | 1 | 1 | 1 | 1 | 1 | 8 |
| Golbasi, Zehra, 2010 | 1 | 1 | 1 | 1 | 1 | 1 | 11 | 8 |
| Hartley, Mary, 2011 | 1 | 1 | 1 | 1 | 2 | 1 | 1 | 8 |
| Heyningen, T. V. 2016 | 1 | 0 | 1 | 1 | 2 | 1 | 1 | 7 |
| Huanging H et al 2017 | 1 | 1 | 1 | 1 | 1 | 1 | 1 | 7 |
| Hu, H. Q. et al 2017 | 1 | 0 | 1 | 1 | 1 | 2 | 1 | 7 |
| Jeong, Hyun-Ghang, et al 2013 | 1 | 1 | 1 | 1 | 1 | 1 | 1 | 8 |
| Kamalak, Z. et al 2016 | 1 | 0 | 1 | 1 | 1 | 1 | 1 | 6 |
| Kaaya SF et al 2009 | 1 | 1 | 1 | 1 | 1 | 1 | 1 | 7 |
| Lara, M. A. et al 2012 | 1 | 1 | 1 | 1 | 1 | 1 | 1 | 7 |
| Lau, Ying, et al 2013 | 1 | 0 | 1 | 1 | 1 | 1 | 1 | 6 |
| Lau, Y. et al 2007 | 1 | 0 | 1 | 1 | 1 | 1 | 1 | 6 |
| Lau, Ying,et al 2011 | 1 | 1 | 1 | 1 | 2 | 1 | 1 | 8 |
| Li, Yingtao, 2016 | 1 | 1 | 1 | 1 | 1 | 1 | 1 | 7 |
| Luna Matos M.L,2009 | 1 | 1 | 1 | 1 | 1 | 1 | 1 | 7 |
| Mahenge, B., 2015 | 1 | 1 | 1 | 1 | 1 | 1 | 1 | 7 |
| Målqvist, M., 2016 | 1 | 0 | 1 | 1 | 2 | 1 | 1 | 7 |
| Manikkam, L. 2012 | 1 | 1 | 1 | 1 | 1 | 1 | 1 | 7 |
| Melo Jr, E. F. 2012 | 1 | 1 | 1 | 1 | 1 | 1 | 1 | 8 |
| Mitsuhiro, S. S. 2009 | 1 | 1 | 1 | 1 | 1 | 1 | 1 | 7 |
| Mohammad, K. I., 2011 | 1 | 1 | 1 | 1 | 2 | 1 | 1 | 8 |
| Moshki, M. et al 2016 | 1 | 1 | 1 | 1 | 1 | 1 | 1 | 8 |
| Mossie, T. B, 2017 | 1 | 1 | 1 | 1 | 1 | 1 | 1 | 8 |
| Nasreen, H. E., 2011 | 1 | 1 | 1 | 1 | 1 | 1 | 1 | 7 |
| Pereira, P. K. 2009 | 1 | 1 | 1 | 1 | 1 | 1 | 1 | 8 |
| Rochat, T. J., et al 2011 | 1 | 1 | 1 | 1 | 1 | 1 | 1 | 7 |
| Rwakarema, M, et al 2015 | 1 | 1 | 1 | 1 | 1 | 1 | 1 | 8 |
| Sahile, M. A. et al 2017 | 1 | 1 | 1 | 1 | 1 | 1 | 1 | 8 |
| Senturk, V. et al , 2011 | 1 | 1 | 1 | 1 | 1 | 1 | 1 | 8 |
| Shakya, R., 2008 | 0 | 1 | 1 | 1 | 1 | 1 | 1 | 6 |
| Shidhaye, P., 2017 | 1 | 1 | 1 | 1 | 1 | 1 | 1 | 7 |
| Silva, Ricardo Azevedo da, 2012 | 1 | 1 | 1 | 1 | 2 | 1 | 1 | 8 |
| Srinivasan, N. 2015 | 0 | 1 | 1 | 1 | 1 | 1 | 1 | 6 |
| Stewart, Robert C.,2014 | 1 | 1 | 1 | 1 | 1 | 1 | 1 | 7 |
| Thompson, O., 2016 | 1 | 1 | 1 | 1 | 2 | 1 | 1 | 8 |
| Waqas, A., 2015 | 1 | 1 | 1 | 1 | 2 | 1 | 1 | 8 |
| Weobong, B. 2014 | 1 | 1 | 1 | 1 | 1 | 1 | 1 | 8 |

- 1. **Longitudinal studies**

|  | **Selection**  (score) | | | | **Comparability**  (score) | | **Outcome**  (score) | | | **Total Score** |
| --- | --- | --- | --- | --- | --- | --- | --- | --- | --- | --- |
| List of studies | Representative of exposed cohort | Selections of non-exposed cohort | Assessment of exposure | Absence of outcome at start of study | Control for Age or Obesity or Smoking or Exercise | Control for other variables (second important variables) | Assessment of outcome | Follow-up period | Adequacy of  follow-up |  |
| Coll, C. D. V. N, 2017 | ★ | ★ | ★ | ★ | ★ | ★ | ★ | ★ | ★ | 8 |
| Guo, N. 2013 | ★ | ★ | ★ | ★ | ★ | ★ | ★ | 0 | 0 | 7 |
| Fisher, J., 2013 | ★ | ★ | ★ | ★ | ★ | ★ | ★ | 0 | 0 | 7 |
| Pottinger, Audrey M.2009 | ★ | ★ | ★ | ★ | ★ | ★ | ★ | 0 | 0 | 7 |
| Padmapriya, N.2016 | ★ | ★ | ★ | ★ | ★ | ★ | ★ | ★ | 0 | 8 |
| Silva, Ricardo, 2012 | ★ | ★ | ★ | ★ | ★ | ★ | ★ | ★ | 0 | 8 |
| Tsai, A. C. 2016 | ★ | 0 | ★ | ★ | ★ | ★ | 0 | ★ | 0 | 6 |
| Vilela, A. A. F. 2014 | ★ | 0 | ★ | ★ | ★ | ★ | 0 | ★ | 0 | 6 |

## Prenatal depression and adverse birth outcomes

- 1. **Longitudinal studies**

|  | **Selection**  (maximum four stars, one for each) | | | | **Comparability**  (maximum two stars, one for each) | | **Outcome**  (maximum three stars, one for each) | | | **Total Score** |
| --- | --- | --- | --- | --- | --- | --- | --- | --- | --- | --- |
| List of studies  (author, date) | Representative of exposed cohort | Selections of non-exposed cohort | Assessment of exposure | Absence of outcome at start of study | Control for Age or Obesity or Smoking or Exercise | Control for other variables (second important variables) | Assessment of outcome | Follow-up period | Adequacy of  follow-up |  |
| Rahman A et al, 2007 | ★ | ★ | ★ | ★ | ★ | ★ | ★ | ★ | 0 | 8 |
| Niemi M et al, 2013 | ★ | ★ | ★ | ★ | ★ | ★ | ★ | ★ | ★ | 9 |
| Sanchez SE et al, 2013 | ★ | ★ | ★ | 0 | ★ | ★ | ★ | ★ | 0 | 7 |
| Chang Hy et al, 2014 | ★ | ★ | ★ | ★ | ★ | ★ | ★ | ★ | ★ | 9 |
| Husain N et al, 2014 | ★ | ★ | ★ | ★ | ★ | ★ | ★ | ★ | 0 | 8 |
| Rao D et al, 2015 | ★ | ★ | ★ | ★ | 0 | ★ | ★ | ★ | 0 | 7 |
| Bindt C et al 2013 | ★ | ★ | ★ | ★ | ★ | ★ | ★ | ★ | 0 | 8 |
| Wado WD et al 2014 | ★ | ★ | ★ | ★ | ★ | ★ | ★ | ★ | ★ | 9 |
| Nasreen HE et al 2010 | ★ | 0 | ★ | ★ | ★ | ★ | ★ | ★ | 0 | 7 |

# **Excluded studies and reason for exclusion**

## Prenatal depression and associated factors

1. Outcome difference (anxiety and depression measurement mixed, perinatal depression) (1-3)
2. Not specific to prenatal depression (CMD, stress, persistent depression throughout pregnancy and postnatal period) (4-11)
3. The study was conducted on restricted population like (adolescent population, disaster affected, HIV positive, hyperemesis gravidarum) (12-18)
4. Postnatal population (19)
5. Depression was not considered as primary outcome variable (20-22)
6. The content of the article was not related to the objective of the current review (letter to the editor, review) (23-28)
7. The content was in Spanish (29)
8. Studies excluded because they had poor quality on NOS (30-36) (<7)

## Effect of prenatal depression on birth outcomes

1. Exposure difference (37-40)
2. Outcome difference (fetal attachment, emergency obstetric as outcome) (41-43)
3. Dissertation abstract for conference (44)
4. Exposure mix and assessment and measurement problem (question used to assess depression) (45-47)
5. The content was in Arabic language (48)
6. Studies excluded because of poor quality on NOS (<7) (49-51)


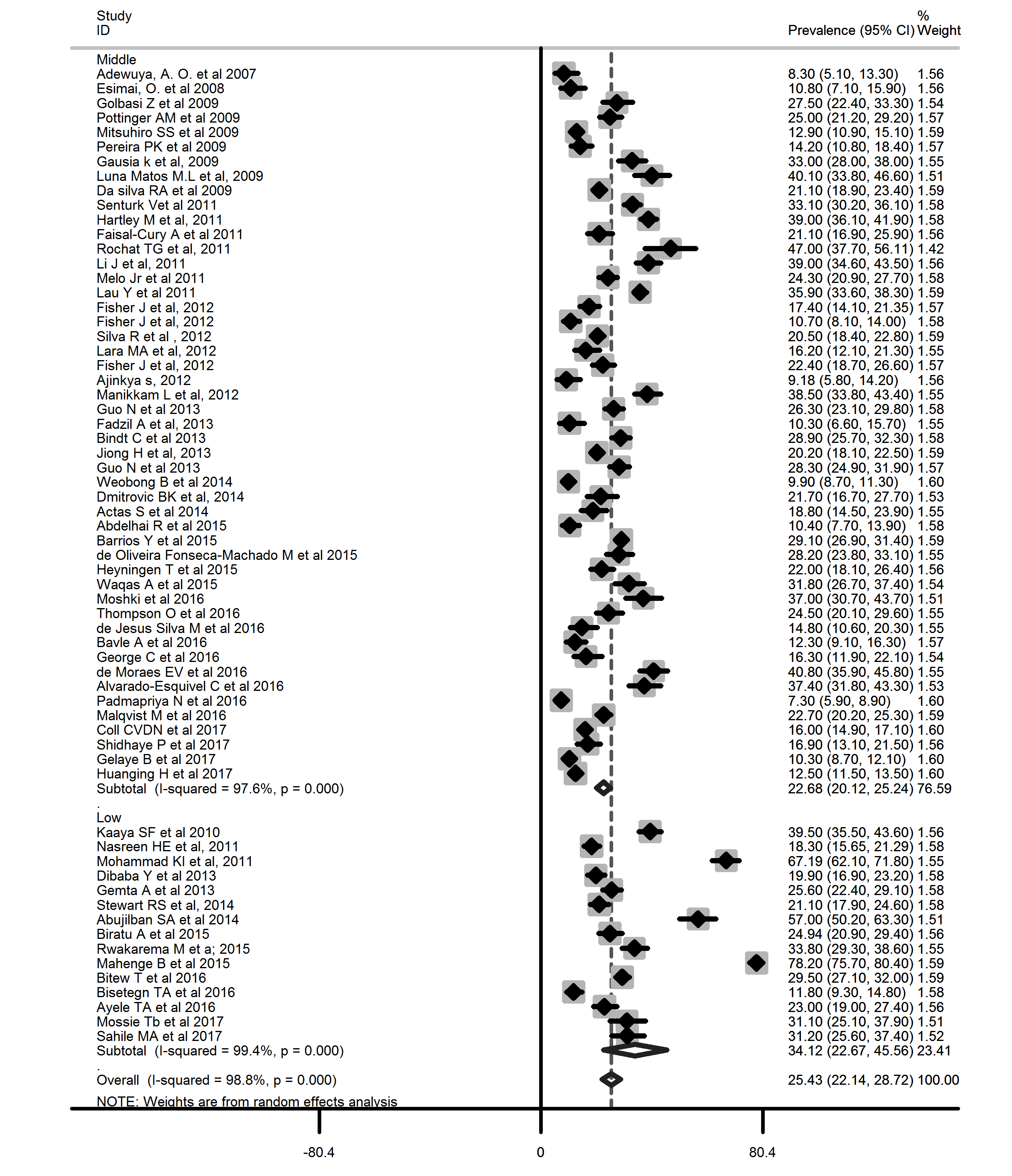


# Fig 1: Meta –analysis of prenatal prevalence sub-analyzed by country income


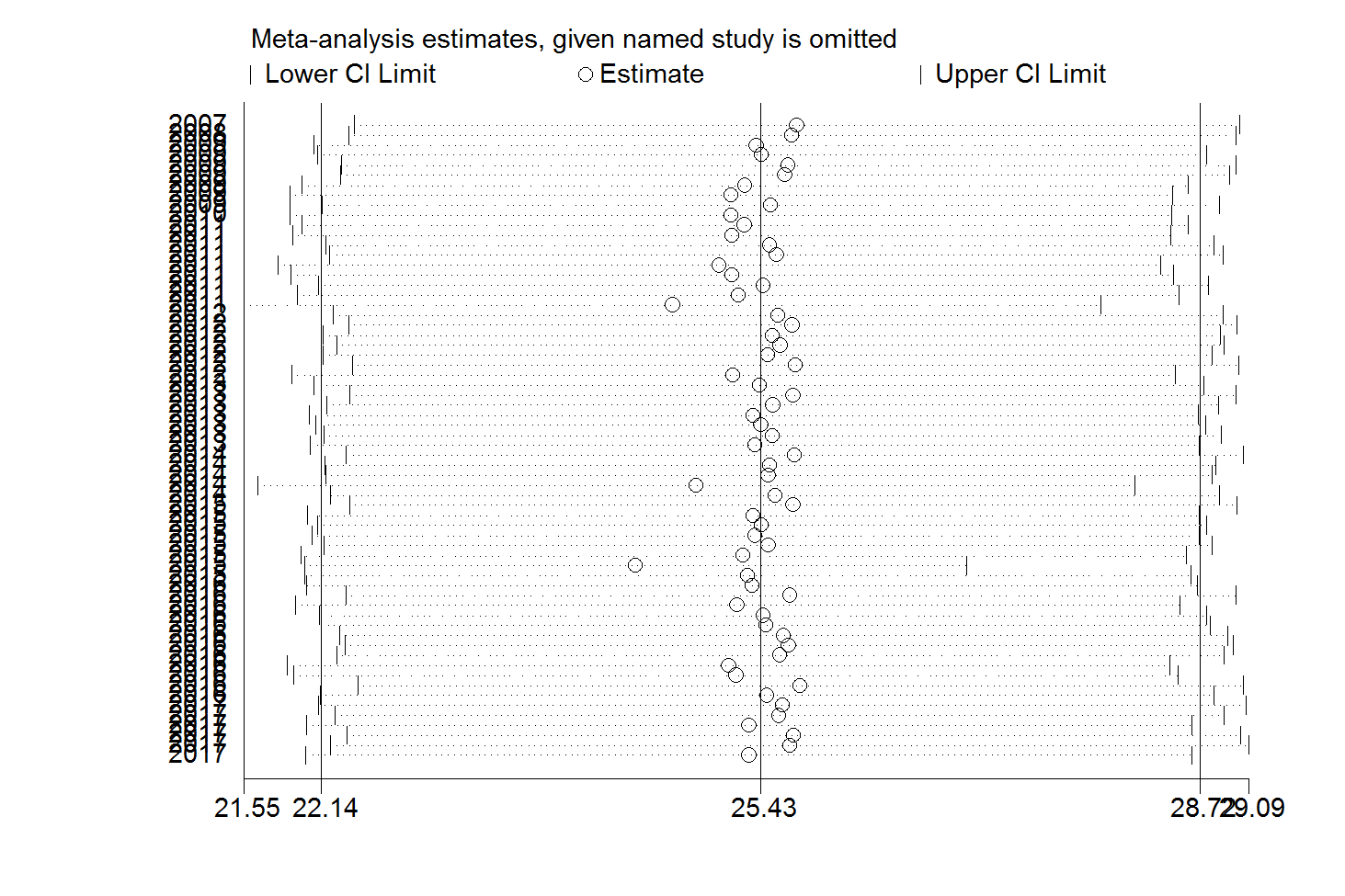


# Fig 6: Sensitivity analysis for prenatal depression prevalence

# **Egger's test for small-study effects**

Number of studies = 64 Root MSE = 8.311

------------------------------------------------------------------------------

Std_Eff | Coef. Std. Err. t P>|t| [95% Conf. Interval]

-------------+----------------------------------------------------------------

slope | .1015521 .0314174 3.23 0.002 .0387496 .1643545

bias | 7.879764 2.171573 3.63 0.001 3.538852 12.22068

**------------------------------------------------------------------------------**

**
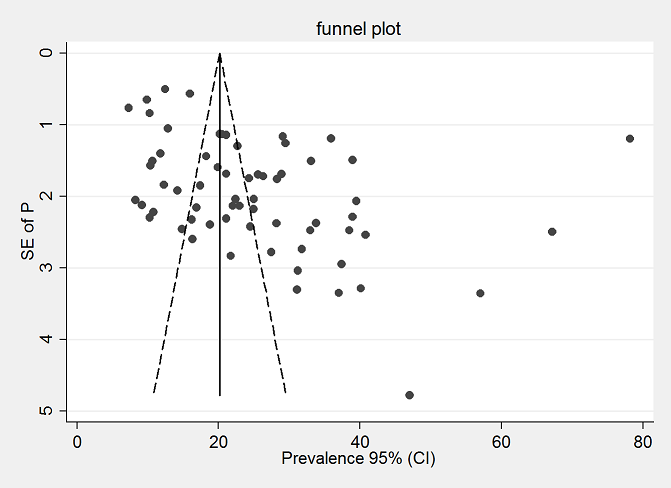
**

# Fig 8: Funnel Asymmetry for testing publication bias on studies of prenatal depression

**
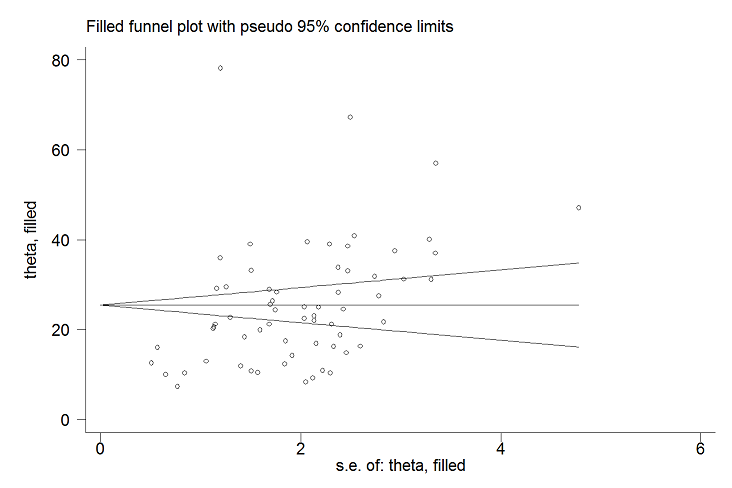
**

# Pooled prenatal depression prevalence after trim and fill analysis

| Pooled 95% CI Asymptotic No. of

Method | Est Lower Upper z_value p_value studies

-------+----------------------------------------------------

Fixed | 20.166 19.812 20.521 111.511 0.000 64

Random | 25.426 22.137 28.716 15.149 0.000

Test for heterogeneity: Q= 5191.627 on 63 degrees of freedom (p= 0.000)

Moment-based estimate of between studies variance = 175.769

# Meta-analysis of factors associated with prenatal depression


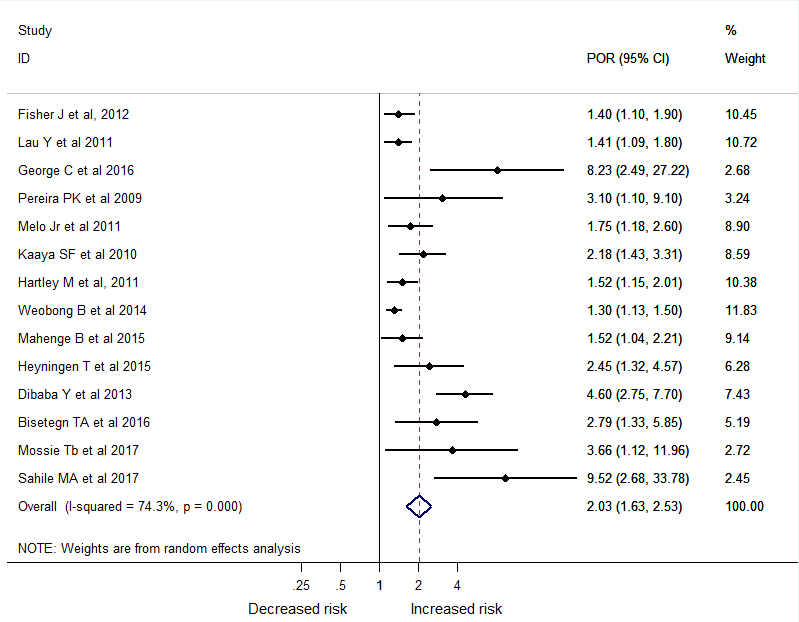


## Fig 9: Economic difficulty as a factor for prenatal depression forest plot


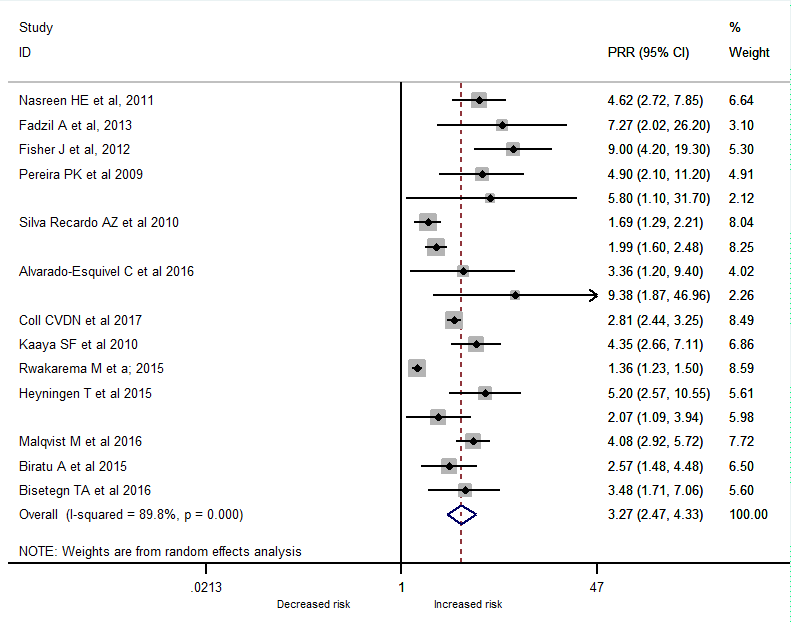


## Fig 9: History of CMD (*depression, anxiety, stressful life event*) as a factor for prenatal depression, forest plot


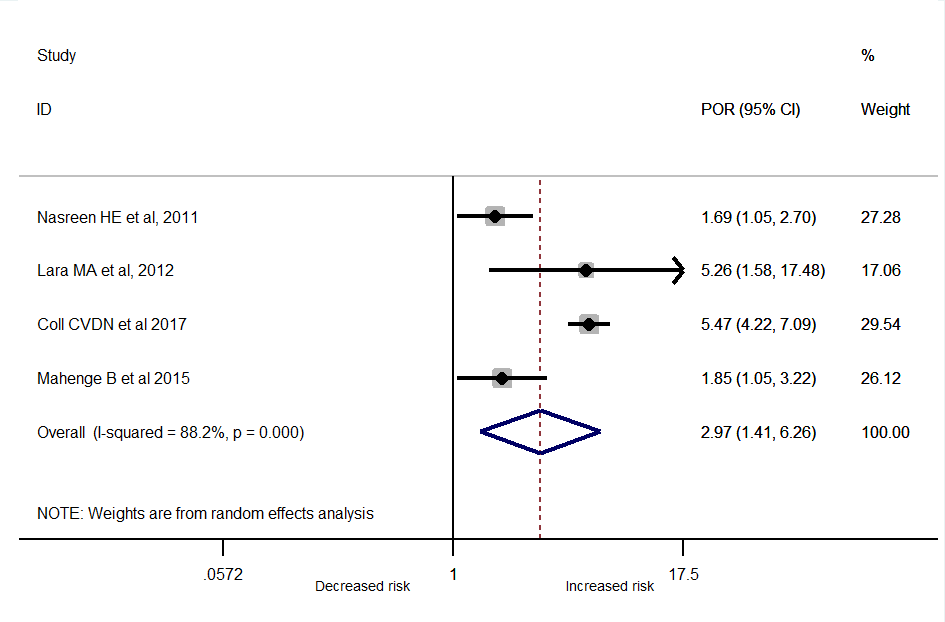


## Fig 10: Male preference in the current pregnancy as a factor for prenatal depression, forest plot


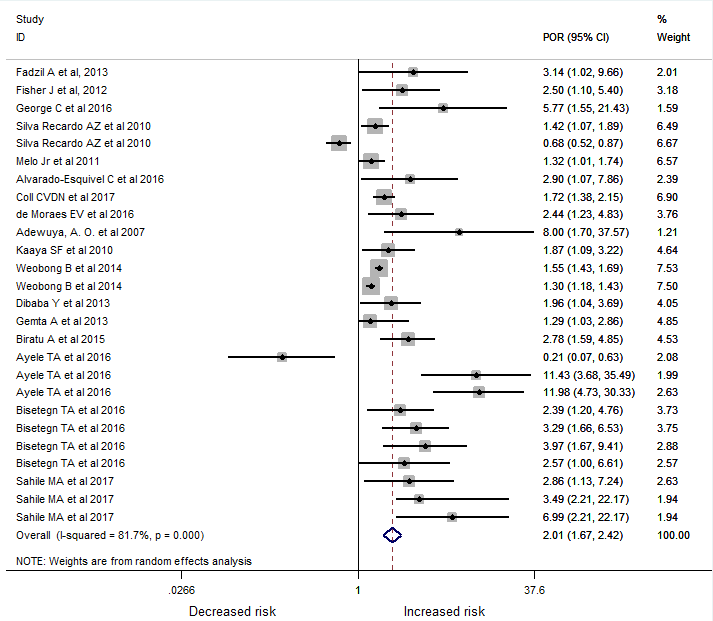


## Fig 11: Bad obstetric history (*unplanned pregnancy, GDM,GHP, labor complication, history of emesis, multiparty*) as a factor of prenatal depression, forest plot


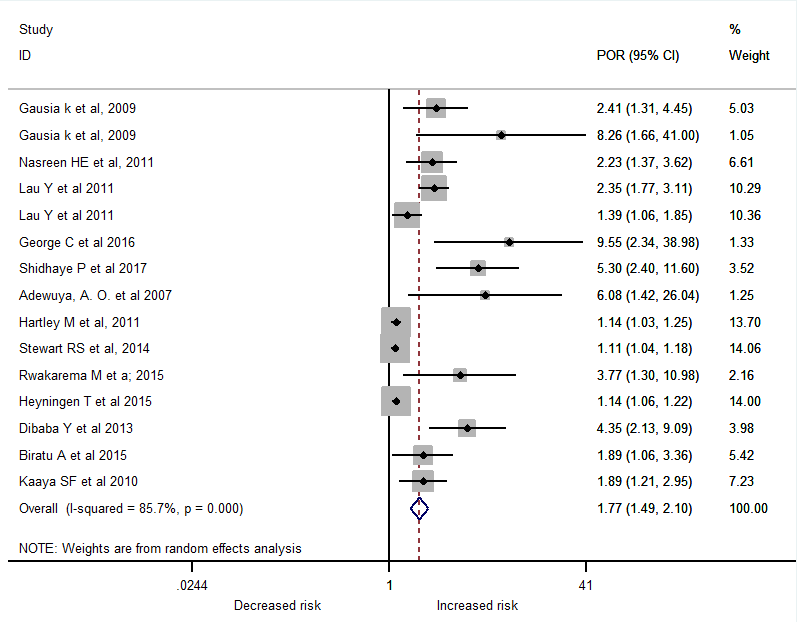


## Fig 12: Poor social support as a risk factor of prenatal depression, forest plot


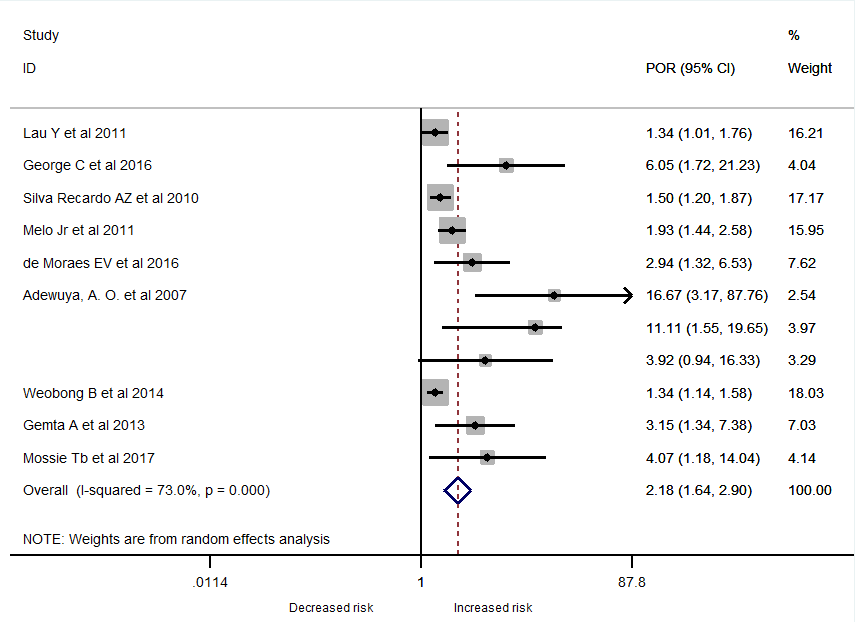


## Fig 13: Unfavorable marital condition as a risk factor for prenatal depression, forest plot


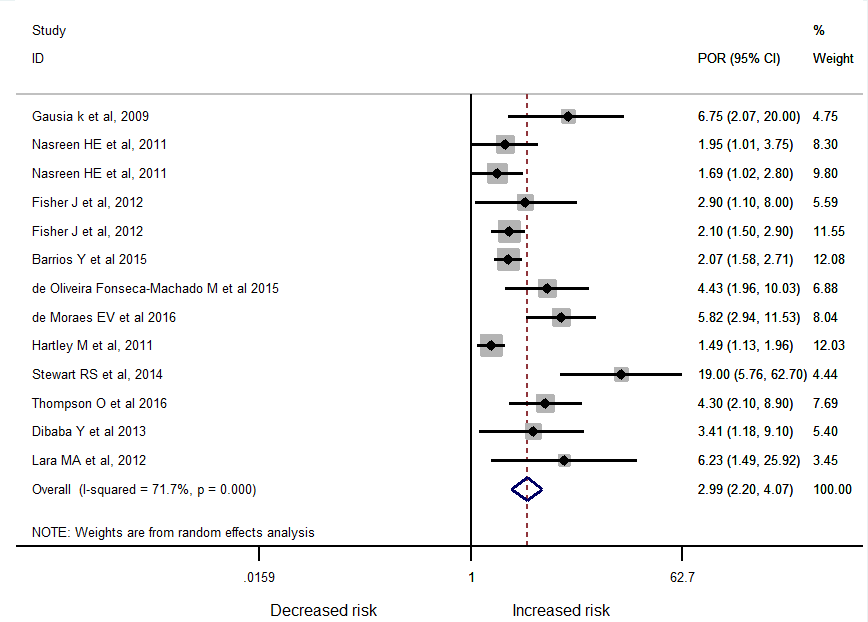


## Fig 14: History of IPV as a risk factor for prenatal depression, forest plot


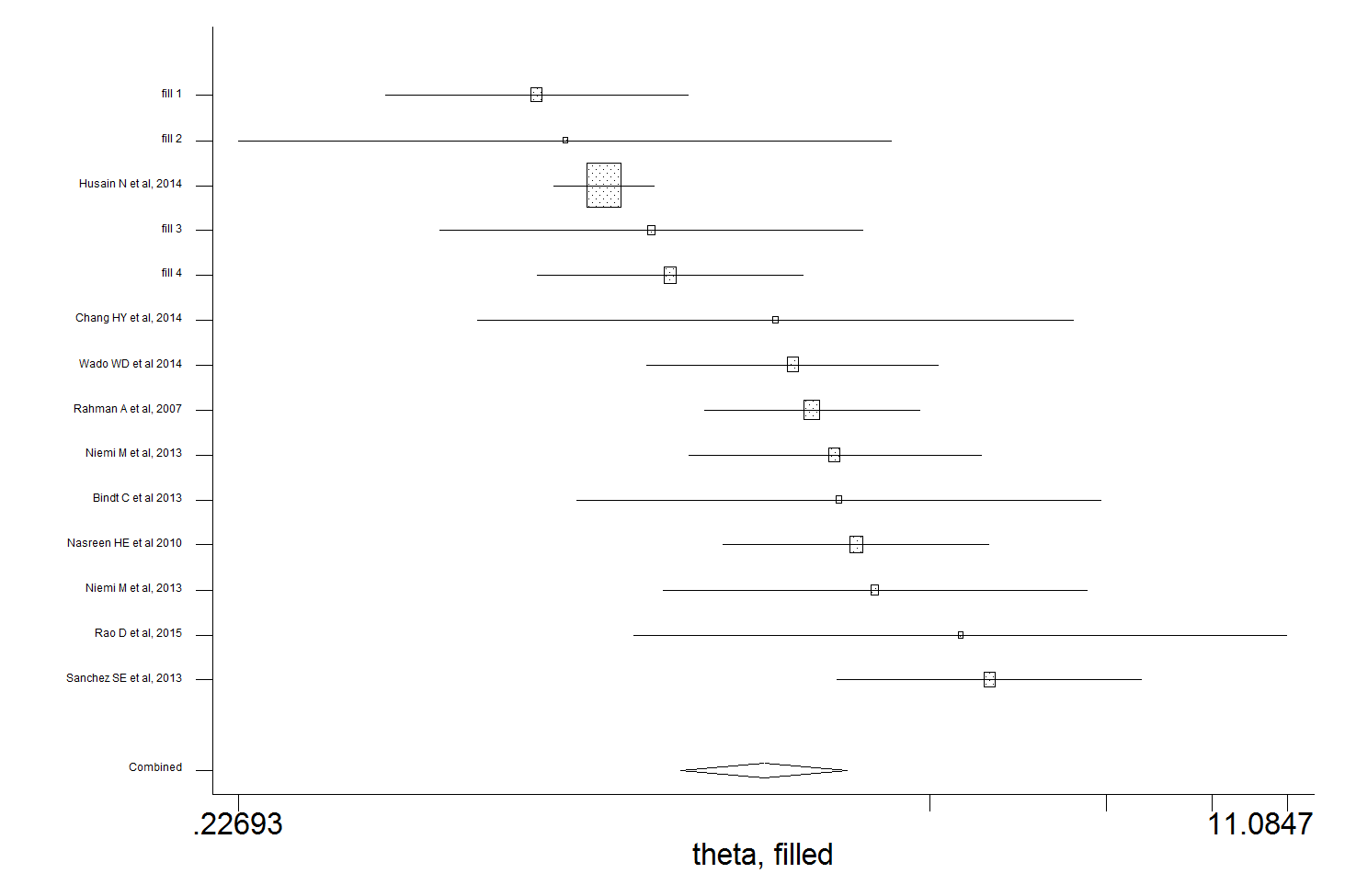


# Fig 16: Forest plot after trim and fill analysis between prenatal depression and adverse birth outcome (four studies filled)

# **Reference**

1. Ali NS, Azam IS, Ali BS, Tabbusum G, Moin SS. Frequency and associated factors for anxiety and depression in pregnant women: a hospital-based cross-sectional study. ScientificWorldJournal. 2012;2012:653098.

2. Karmaliani R, Asad N, Bann C, Moss N, Mcclure EM, Pasha O, et al. Prevalence of anxiety, depression and associated factors among pregnant women of Hyderabad, Pakistan. Int J Soc Psychiatry. 2009;55.

3. Tan EC, Chua TE, Lee TMY, Tan HS, Ting JL, Chen HY. Case-control study of glucocorticoid receptor and corticotrophin-releasing hormone receptor gene variants and risk of perinatal depression: BMC Pregnancy and Childbirth. 15 (1) (no pagination), 2015. Article Number: 283. Date of Publication: October 30, 2015.; 2015.

4. Almeida CP, Cunha FF, Pires EP, SÁ E. Common mental disorders in pregnancy in the context of interpartner violence. Journal of Psychiatric & Mental Health Nursing. 2013;20(5):419-25.

5. Carolan-Olah M, Barry M. Antenatal stress: An Irish case study: Midwifery. 30 (3) (pp 310-316), 2014. Date of Publication: March 2014.; 2014.

6. Faisal-Cury A, Menezes P, Araya R, Zugaib M. Common mental disorders during pregnancy: prevalence and associated factors among low-income women in Sao Paulo, Brazil: depression and anxiety during pregnancy. Archives of Women's Mental Health. 2009;12(5):335-43.

7. Fisher J, Tran T, La BT, Kriitmaa K, Rosenthal D. Common perinatal mental disorders in northern Viet Nam: community prevalence and health care use. Bulletin of the World Health Organization. 2010;88(10):737-45.

8. Khan AM, Flora MS. Maternal common mental disorders and associated factors: A cross-sectional study in an urban slum area of Dhaka, Bangladesh. International Journal of Mental Health Systems. 2017;11(1).

9. Ludermir AB, Valongueiro S, Araujo TV. Common mental disorders and intimate partner violence in pregnancy. Revista de Saude Publica. 2014;48(1):29-35.

10. Rahman A, Creed F. Outcome of prenatal depression and risk factors associated with persistence in the first postnatal year: prospective study from Rawalpindi, Pakistan. Journal of Affective Disorders. 2007;100.

11. Uguz F, Gezginc K, Kayhan F, Sar S, Buyukoz D. Is pregnancy associated with mood and anxiety disorders? A cross-sectional study: General Hospital Psychiatry. 32 (2) (pp 213-215), 2010. Date of Publication: March 2010.; 2010.

12. AsunciÃ³n Lara Ma, Berenzon S, JuÃ¡rez GarcÃ­a F, Medina-Mora MaE, Natera Rey G, Ameth Villatoro VelÃ¡zquez J, et al. Population study of depressive symptoms and risk factors in pregnant and parenting Mexican adolescents. Revista Panamericana de Salud Publica. 2012;31(2):102-8.

13. Lau Y, Yin L, Wang Y. Severe antenatal depressive symptoms before and after the 2008 wenchuan earthquake in chengdu, china. JOGNN: Journal of Obstetric, Gynecologic & Neonatal Nursing. 2011;40(1):62-74.

14. Lukose A, Ramthal A, Thomas T, Bosch R, Kurpad A, Duggan C, et al. Nutritional Factors Associated with Antenatal Depressive Symptoms in the Early Stage of Pregnancy Among Urban South Indian Women. Maternal & Child Health Journal. 2014;18(1):161-70.

15. Matos MLL, Pielago JS, Figueroa AL. Major depression in pregnant women served by the National Materno-Perinatal Institute in Lima, Peru. [Spanish]: Revista Panamericana de Salud Publica/Pan American Journal of Public Health. 26 (4) (pp 310-314), 2009. Date of Publication: October 2009.; 2009.

16. Rochat T, Tomlinson M, Newell M, Stein A. Depression among pregnant women testing for HIV in rural South Africa: Implications for VCT. 9th International AIDS Impact Conference; Botswana2009.

17. Tan PC, Zaidi SN, Azmi N, Omar SZ, Khong SY. Depression, anxiety, stress and hyperemesis gravidarum: Temporal and case controlled correlates. PLoS ONE Vol 9(3), 2014, ArtID e92036. 2014;9(3).

18. Ying Z, Kane I, Liping M, Shenxun S, Jing W, Qiping L, et al. The Prevalence of Antenatal Depression and its Related Factors in Chinese Pregnant Women who Present with Obstetrical Complications. Archives of Psychiatric Nursing. 2016;30(3):316-21.

19. Bodhare TN, Sethi P, Bele SD, Gayatri D, Vivekanand A. Postnatal Quality of Life, Depressive Symptoms, and Social Support Among Women in Southern India. Women & Health. 2015;55(3):353-65.

20. Farias DR, Pinto Tde J, Teofilo MM, Vilela AA, Vaz Jdos S, Nardi AE, et al. Prevalence of psychiatric disorders in the first trimester of pregnancy and factors associated with current suicide risk. Psychiatry Research. 2013;210(3):962-8.

21. Qiu C, Sanchez SE, Lam N, Garcia P, Williams MA. Associations of depression and depressive symptoms with preeclampsia: Results from a Peruvian case-control study: BMC Women's Health. 7 (no pagination), 2007. Article Number: 15. Date of Publication: 27 Sep 2007.; 2007.

22. Rahman A, Bunn J, Lovel H, Creed F. Association between antenatal depression and low birth weight in a developing country. Acta Psychiatr Scand. 2007;115.

23. Glover V. Maternal depression, anxiety and stress during pregnancy and child outcome; what needs to be done. Best Practice & Research Clinical Obstetrics & Gynaecology. 2014;28(1):25-35.

24. Jokhi R. Antenatal depression: how midwives can help. Practising Midwife. 2013;17(5):21-3.

25. Lee HH, Kim TH. Screening depression during and after pregnancy using the EPDS. Archives of Gynecology and Obstetrics. 2014;290(4):601-2.

26. Leung BM, Kaplan BJ. Perinatal depression: prevalence, risks, and the nutrition link--a review of the literature. Journal of the American Dietetic Association. 2009;109(9):1566-75.

27. Marcus S, Lopez JF, McDonough S, MacKenzie MJ, Flynn H, Neal Jr CR, et al. Depressive symptoms during pregnancy: Impact on neuroendocrine and neonatal outcomes. Infant Behavior and Development. 2011;34(1):26-34.

28. Sanghera R, Wong ST, Brown H. A cross-sectional analysis of perinatal depressive symptoms among Punjabi-speaking women: are they at risk? BMC Pregnancy & Childbirth. 2015;15:151.

29. Ricardo-Ramirez C, Alvarez-Gomez M, Ocampo-Saldarriaga MV, Tirado-Otalvaro AF. Prevalence of positive screening for depression and anxiety in high obstetric risk pregnant women in a clinic in Medellin, Colombia, between January and August 2013, and associated risk factors. [Spanish]: Revista Colombiana de Obstetricia y Ginecologia. 66 (2) (pp 94-102), 2015. Date of Publication: 2015.; 2015.

30. Kamalak Z, Kosus N, Kosus A, Hizli D, Akcal B, Kafali H, et al. Adolescent pregnancy and depression: is there an association? Clinical & Experimental Obstetrics & Gynecology. 2016;43(3):427-30.

31. Lau Y. Risk factors associated with antenatal depressive symptomatology among Chengdu Chinese women. Merrick, Joav [Ed] (2013) Alternative medicine yearbook, 2011 (pp 107-121) xxx, 572 pp Hauppauge, NY, US: Nova Biomedical Books; US. 2013:107-21.

32. Lau Y, Keung DW. Correlates of depressive symptomatology during the second trimester of pregnancy among Hong Kong Chinese. Social Science & Medicine. 2007;64(9):1802-11.

33. Shakya R, Situala S, Shyangwa PM. Depression during pregnancy in a tertiary care center of eastern Nepal. Jnma, Journal of the Nepal Medical Association. 2008;47(171):128-31.

34. Srinivasan N, Murthy S, Singh AK, Upadhyay V, Mohan SK, Joshi A. Assessment of burden of depression during pregnancy among pregnant women residing in rural setting of chennai. Journal of Clinical and Diagnostic Research JCDR. 2015;9(4):LC08-12.

35. Tsai AC, Tomlinson M, Comulada WS, Rotheram-Borus MJ. Food insufficiency, depression, and the modifying role of social support: Evidence from a population-based, prospective cohort of pregnant women in peri-urban South Africa. Social Science & Medicine. 2016;151:69-77.

36. Vilela AAF, Farias DR, Eshriqui I, Vaz JdS, Franco-Sena AB, Castro MBT, et al. Prepregnancy healthy dietary pattern is inversely associated with depressive symptoms among pregnant Brazilian women. Journal of Nutrition. 2014;144(10):1612-8.

37. Bałkowiec-Iskra E, Mirowska-Guzel DM, Wielgoś M. Effect of antidepressants use in pregnancy on foetus development and adverse effects in newborns. Ginekologia Polska. 2017;88(1):36-42.

38. Ferri CP, Mitsuhiro SS, Barros MCM, Chalem E, Guinsburg R, Patel V, et al. The impact of maternal experience of violence and common mental disorders on neonatal outcomes: A survey of adolescent mothers in Sao Paulo, Brazil. BMC Public Health. 2007;7.

39. Stewart RC, Umar E, Kauye F, Bunn J, Vokhiwa M, Fitzgerald M, et al. Maternal common mental disorder and infant growth--a cross-sectional study from Malawi. Matern Child Nutr. 2008;4.

40. Khatun S, Rahman M. Socio-economic determinants of low birth weight in Bangladesh: A multivariate approach. Bangladesh Medical Research Council Bulletin. 2008;34(3):81-6.

41. Diniz E, Volling BL, Koller SH. Social support moderates association between depression and maternal-fetal attachment among pregnant Brazilian adolescents. Journal of Reproductive and Infant Psychology. 2014;32(4):400-11.

42. Rahman A, Creed F. Outcome of prenatal depression and risk factors associated with persistence in the first postnatal year: prospective study from Rawalpindi, Pakistan. J Affect Disord. 2007;100(1-3):115-21.

43. Bayrampour H, Salmon C, Vinturache A, Tough S. Effect of depressive and anxiety symptoms during pregnancy on risk of obstetric interventions. Journal of Obstetrics & Gynaecology Research. 2015;41(7):1040-8.

44. Nylen KJ. Effects of prenatal maternal distress on reproductive outcomes. Dissertation Abstracts International: Section B: The Sciences and Engineering. 2010;70(8-B):5178.

45. Qiao Y, Wang J, Li J, Wang J. Effects of depressive and anxiety symptoms during pregnancy on pregnant, obstetric and neonatal outcomes: a follow-up study. Journal of Obstetrics & Gynaecology. 2012;32(3):237-40.

46. Smith KF, Huber LRB, Issel LM, Warren-Findlow J. The Association Between Maternal Depression During Pregnancy and Adverse Birth Outcomes: A Retrospective Cohort Study of PRAMS Participants. Journal of Community Health. 2015;40(5):984-92.

47. Gungor I, Oskay U, Beji NK. Biopsychosocial risk factors for preterm birth and postpartum emotional well-being: a case-control study on Turkish women without chronic illnesses. J Clin Nurs. 2011;20(5-6):653-65.

48. Neshat R, Majlesi F, Rahimi A, Shariat M, Pourreza A. Investigation the relationship between preterm delivery and prevalence of anxiety, stress and depression in pregnant women of dorrod health center, Iran in 2010. [Persian]: Iranian Journal of Obstetrics, Gynecology and Infertility. 16 (67) (pp 16-24), 2013. Date of Publication: September 2013.; 2013.

49. Kim HW, Jung YY. [Effects of antenatal depression and antenatal characteristics of pregnant women on birth outcomes: a prospective cohort study]. Journal of Korean Academy of Nursing. 2012;42(4):477-85.

50. Saeed A, Raana T, Saeed AM, Humayun A. Effect of antenatal depression on maternal dietary intake and neonatal outcome: a prospective cohort. Nutrition Journal. 2016;15(1):64.

51. Yang S, Yang R, Liang S, Wang J, Weaver NL, Hu K, et al. Symptoms of anxiety and depression during pregnancy and their association with low birth weight in Chinese women: A nested case control study. Archives of Women's Mental Health. 2017;20(2):283-90.
