# Supplementary material for: Antenatal depression and its association with adverse birth outcomes in low and middle-income countries: A systematic review and meta-analysis
Source: PLoS One. 2020 Jan 10;15(1):e0227323. doi: 10.1371/journal.pone.0227323 (PMC6953869; doi:10.1371/journal.pone.0227323)
Supplement: Attachment — Submitted filename: Rebuttal letter.docx [file pone.0227323.s002.docx]

**Date 25/10/2019**

**Rebuttal letter for reviewers**

**Article title:** Prenatal Depression and its Effect on Birth Outcomes in Low and Middle-income Countries: A Systematic Review and Meta-analysis (PONE-D-19-25076)

**Corresponding author:** Abel Fekadu Dadi

Dear reviewers and editor;

Thank you for your time and effort to bring the status of this manuscript to the standard. We have made all necessary revisions based on reviewers and editor comments and suggestions. We have presented reviewers comment and authors response using the next table.

| **Editor comment** | **Author response** |
| --- | --- |
| Please provide any updates you might have since the original search was performed in XXX, or please provide the rational for ending your search at that time. | We haven’t made any change since the original search. This systematic review has been registered in Prospero and the date specified was the recent one at that time. |
| **Reviewer comment** |  |
| **Reviewer #1** |  |
| Thank you for the opportunity to review this interesting and informative piece. I found, at times, there were minor edits required especially in the abstract. Overall I found the paper well considered, referenced, and articulated. I strongly recommend the authors follow a standard format for their reference list - it is a mixture of different formats which is distracting and indicates an editing issue. | Thanks reviewer. We have fixed the reference issue in the new manuscript. |
| Personally i would appreciate a visual (such as a flow chart) of article identification, fit with criteria, etc. | Thanks reviewer for your keen observation. The PRISMA diagram, table, the quality assessment process, and some of the analysis results were included in separate supplementary information, which separately uploaded during the submission. I would respectfully refer you to this submission for your reference. |
| **Reviewer #2** |  |
| Abstract The authors loosely described the gap of the research.  The authors also need to describe the statistical issues in this section. | Thanks reviewer for your keen observation.  We shortly and clearly put the gap in information that affects antenatal depression not a priority issue as you know abstract is subjected to limited word count. We revised a little bit. Depression in pregnancy (antenatal depression) in many low and middle-income countries is not well documented and has not been given priority for intervention due to competing urgencies and the belief that it does not immediately cause fatalities, which mainly emanated from lack of comprehensive research on the area. To fill this research gap, this systematic review was conducted to investigate the burden of antenatal depression and its consequences on birth outcomes in low- and middle-income countries.”    Thanks reviewer. We believe that points in statistical issues are well described shortly. However, we are keen to know and include issues that you are really interested in. |
| The authors did not follow the PRISMA checklist for this systematic review. I recommend them to revise based on this systematic review is based on the Preferred Reporting Items of Systematic Reviews and Meta-Analysis (PRISMA) checklist guidelines to ensure scientific rigor. This systematic review lacks format especially in the method section.  ]  The authors also did not mention several subtitled for what they are mentioned in the method section. (Information sources); Authors did not include other major databases such Hinari?    Authors did not report anything about the grey literature that also needs to be explained here. Newcastle-Ottawa Scale is over simplified, and authors should also consider NIH scale for observational studies as it is more detailed one to assess quality. | Thanks reviewer. Yes, you are wright. The PRISMA diagram, the table or checklists, quality assessment results and other some stastical analysis results were attached as a supplementary information to meet the journal guideline and I respectfully refer you to this document to be sure that all aspects of the PRISMA are fulfilled.  Thanks ones again. In the method section of this article, under search strategy we clearly put the data sources of the articles like this “CINHAL, MEDLINE, EMCare, [PubMed](https://www.ncbi.nlm.nih.gov/pubmed?otool=iauflullib&myncbishare=flinlib), Psych Info [and Scopus](http://ezproxy.flinders.edu.au/login?url=https://www.scopus.com/scopus/home.url) data bases were systematically searched for the following key terms: Pregnan*, antenat*, depression, clinical depression, depressed mood, major depressive disorder, depressive symptom, adverse birth outcomes, stillbirth, preterm birth, and low birth weight. Studies were excluded if a standardized approach was not used to measure main outcomes.”  To be clear this data bases are the main databases that contain mental health articles more than any databases and were selected because of this. HINARI is a data base developed by WHO for low income countries to access articles free of charge and for my case because of my university, Flinders university, I could have accessed all articles for free because of their organized resource facilities for students. All the searching processes were supported by well equipped librarians of the university.  Thank you, reviewer, for your insight. We did not include grey literatures, we missed to include under this section. I included in the revised version. Newcastle-Ottawa Scale is the most reputable tool for quality assessment for observational study and is also as detailed as NIH, I do not see much deference in detailing and I searched a reference for this and not found one. NIH is much of internal quality and limited in presenting an objective measurement to finally rate the article as good or poor and it solely left for researchers’ judgement. More, importantly, we believe that NOS is also appropriate and complete to use for quality assessment so far. |
| **Result section** Throughout the manuscript the authors need to use the journal preparation guideline while citing the figures.  Page 5, paragraph 2 and 1 line, two independent reviewers … the authors need to mention who these authors are? Apply this comment also throughout the manuscript.  On the same paragraph, line the authors considered good quality articles according to Newcastle Ottawa Scale greater than 7. What is your reference to use 7 as a cut-off point?  Page 6, the authors described that 46% of the included studies were health institutional based studies while the rest are community. So, why authors merge the two different setting studies? Are they similar population? How do you see the PICO criteria? | Received. We have revised in the newly submitted version. Thanks  Revised now. Thanks  Thanks reviewer. The reference was included on the first line of the paragraph.  Thank you, reviewer, for your keen observation. We did the pooling in two steps. Over all pooling, to see the extent of the problem and we also did a sub-analysis. One of the sub-analysis was made between different study settings. (see table 5) |
| The authors described that they performed a subgroup analysis based on the publication year. 1) What is the advantage of reporting subgroup analysis based on publication year? I recommend the authors to report study period than publication year. Some articles published within one year of its done while others last 2/3/4/5 and more year that means a study conducted in 2010 may be published in 2013. Similarly, what about publication between 2012 to 2014?  2) What is the base for categorizing publication year as 2011-2012 and 2015-2016? I need strong justification on this. | Thank you, reviewer, for your keen observation. We believed and literatures agree on date of publication than the date at which the study was conducted as a standard or as consensus. And we followed this based on the time at which the information is available online. The subdivision was based on two years gap just to superficially show the trend over time though many factors might affect this. It has been included in one of these categories; 2011- 2012, 2013-2014.  The justification to subdivided in two years gap was just two narrow the category and simply show the trend over time. It is a consensus issue between all outers to present like this and there was no as such a hard and soft rule for it. It is was also possible to present it in yearly based but it would take space. |
| Discussion Generally the authors need to improve more this section. All the articles that present the results of an investigation have to end in a section of discussion of results and conclusions. Therefore better discussion sounds reader and increase the quality of the study. These sections of the article should not be confused as they answer three clear questions, what was found? For the Results section, what is the meaning of what I found? When we are referring to the Discussion section and what are the most important findings of our work? Specifically Page #9, the authors mentioned that one of the reason for the increased postnatal depression is number of studies conducted in the setting. How could this be a reason for the increment?    Page #9, first line of last paragraph, the authors reported that prenatal depression prevalence increases from the first to the third trimester of pregnancy which also contradict with previous study reports. What did the authors think for potential justification for this increment? The authors also did not mention any possible reason, justification for their findings. I strongly recommend also the authors to state the possible justification for each contradicting findings and also the implication of the findings.    Page #10, last paragraph, I am not clear about this paragraph. The paragraphs describe about evidences taken from other source. The authors did not mentioned what happened on their review about social support. Did the authors assessed the effects of social support on prenatal depression? If so what was the relationship?  On the same paragraphs last statement has no source? | Sure, and we believe that we have presented in such way.  Thanks reviewer. These are the two  hypothesises that everybody could pose for the increment of the prevalence overtime. The real increase of the problem overtime might be because of its lack of priority or the problem has been well researched by researchers over time and they brought the real burden of the disorder. This is true that a number of prevalence studies on antenatal depression are significantly published in the last three years in Ethiopia. These two hypotheses can be evidenced in the table that presented a sub-analysis on year of publication. Number of studies were increased over time and where the number of studies were high the prevalence was also high. We have revised this section to clearly describe this hypothesis.  Thanks, this is a review and not primary study so that the reason for contradiction might be personal judgement. But in general, the well-known hypothesis was the parabolic one (high during the 1^st^ trimester, low in the second and start to increase in the third trimester). Antenatal depression is high during the first trimester as biologically indicated that hormonal secretions following the pregnancy increases her stress hormone production and emotional sickness that emerge during this time put the mothers in the state of depression. In the mid -pregnancy the mothers already familiar with these biological changes and her motion sicknesses already gone and she becomes in stable state of mind. However, when she approaches delivery, she starts to worry about the fate of her pregnancy, birth outcomes, place of delivery and consequences, which again increases her stress level and make her depressed. But in our review, the prevalence appeared to increase over the trimesters, this could be related to the number of studies included in the review, from the result we could see that as the number of included studies increase the prevalence was also increased. But now a days, including this review, one other review also reported an incremental nature of depression over the trimesters which needs further investigation as we recommended. However, I have made revision on the justification.  Thanks, it is true that social support is a social buffer of stressors meaning that mothers with social support has low risk of developing depression and this sentence has been shortly indicated. I made the sentence complete like this in the revised manuscript.  Thanks again for your intention to make the content clearer. Here I made it clear. It is just to mean that proper nutrition during pregnancy prevents the risk of antenatal depression, however, mothers living in low income countries are living under economic stress which also indirectly affects their risk of having depression during pregnancy. |
| Limitation The author described somewhere else in the method section that they included articles that assessed prenatal depression using standard measured or standardized approach using validated screening tools. However, the authors mentioned measurement bias as a limitation. How could this go together? What did the authors did to overcome this limitation?  Reference The authors describe somewhere else in the method section that they managed the reference using EndNote software. However, most references listed under the referral list is not correct and does not fulfill the journal requirement. Therefore, I strongly recommend the authors to revise the EndNote library and also correct the reference list. Example: look reference #2, consider others also.  Figure Figure 2, delete the weight of individual /primary studies. What is PRR mean in the figure? In forest plots; only surname of authors will be enough, and studies should be either in alphabetic order or in year of publication order. Revise all. | Thanks reviewer. This limitation indicates the general limitation of using screening instruments over diagnostic tools as they are not as valid as diagnostic tools such as DSM and ICD. We tried to minimize this limitation by including only primary studies that used validated screening tool. This is already highlighted in this section.  Thank you very much. I have addressed all the reference issues.  Thanks. We believe that the presence of the weight that each individual study contributed to the pooled effect should be presented as it further adds an information to the forest plot. Similarly, putting only surname shows that the author is a sole author of the study. The order of the studies has been revised in order of publication year. |
