# Supplementary material for: Antenatal depression and its association with adverse birth outcomes in low and middle-income countries: A systematic review and meta-analysis
Source: PLoS One. 2020 Jan 10;15(1):e0227323. doi: 10.1371/journal.pone.0227323 (PMC6953869; doi:10.1371/journal.pone.0227323)
Supplement: Attachment — Submitted filename: rebuttal.docx [file pone.0227323.s003.docx]

**Date 04/12/2019**

**Rebuttal letter for editor comment**

**Article title:** Prenatal Depression and its Effect on Birth Outcomes in Low and Middle-income Countries: A Systematic Review and Meta-analysis (PONE-D-19-25076)

**Corresponding author:** Abel Fekadu Dadi

Dear editor;

Thank you for your time and effort to bring the status of this manuscript to the standard. We have made all necessary revisions based on your comments and suggestions and presented a point by point response using the next table.

| **Editor comment** | **Author response** |
| --- | --- |
| Introduction • Please update all old references used in the introduction section. For example, in 2015, the global prevalence of depression was estimated to be 4.4%. | Thanks editor;  I have revised evidences that have updated literatures. |
| Methods • On your data sources (searching engine), why you did not search from EMBASE, which is a large database. | Thanks. Yes, EMBASE and MEDLINE are the biggest databases for health researches but depends on a specific topic of your study. We used journal database predictions to identify databases where mental health issues are mostly published. So, unfortunately, in my case, MEDLINE was preferred than EMBASE. |
| Besides, you systematic review focused on depression. So, why not you searched from PsycINFO? | Psych INFO was the main database and I used it and it was there. But the way I wrote it was wrong and corrected it now. |
| Why you restricted your period from January 1, 2007 and December 31, 2017? | Thanks.  I used 10 years span of search to just get the most updated literature on the area. |
| I saw your search strategy from additional files, but I am not happy because it was not detail and compressive. | I am not clear in terms of what? Maybe I did not put search strategies for all data bases but they were almost similar unless the trickies added to the main terms in specific databases. |
| • Who are high risk? | Thank you for your keen observation.  I should have made it specific and we considered studies conducted on the following restricted populations or high risk populations for depression: studies conducted in refugee camps, conducted following certain disasters, mothers living with HIV/TB etc, conducted on primi-mothers or women with pregnancy complications etc as we hypothesised that apart from sub-analysis, estimates come from such studies would not clearly represent the actual source population. |
| • Could you attached your NOS tool because as far I know NOS has 10 maximum points? | Sorry for the editorial issues.  Yes, NOS contains ten points with 7 has been used as a cut of value for good quality article but it depends on the design. For cross-sectional 10 points maximum, for cohort 9 stars maximum, and it is also different for case control studies. The whole quality assessment points for all articles included in the study has been described in supplementary document starting from page six. |
| • How you decided the category of quality scores as good, fair and poor? | Based on the points, there is standard for that for each specific study design. This is included under risk of bias.  The NOS includes 3 categorical criteria with a maximum score of 10 points: “selection” which accounts a maximum of 5 points, “comparability” which accounts a maximum of 2 points, and “outcome” which accounts a maximum of 3 points. The quality of each study was rated using the following scoring algorithm: ≥7 points was considered as “good” quality study, 2 to 6 points was considered as “fair” quality study, and ≤ 1 point was considered as “poor” quality study. |
| • You reported a meta-analysis of proportions for antenatal depression. Therefore, for proportion or prevalence meta-analysis, the appropriate command is metaprop instead of metan command. Metan command is used to estimate the effect. Could you revise your analysis based on the above suggested command? | Thank you, editor. Metaprop command is used when you have row data or binomial data, means, if you have number of individuals with the condition and the number of the whole population included in the study. Assuming that there is no study with proportions near to the extremities like 0 and 1 you can use metan command as well. My prevalence data is normally distributed and it was possible to use the metan command. The data you require is the estimate which is proportion or prevalence and its confidence interval.  *Metan proportion lci uci*  <https://archpublichealth.biomedcentral.com/articles/10.1186/2049-3258-72-39> |
| • For publication biases, objective tests (Egger's and Begg's tests). Therefore, please remove funnel plot. | Thanks. I would prefer to present both because of the following reasons. There is additional information that you got using funnel plot over the objective test. The objective tests just give you a p-value but the funnel plot shows you the characteristics of included studies and the characteristics of the missed studies: are they big studies, small studies, or if the dispersion is caused from the quality of the included studies. It can also tell you the number and characteristics of studies filled by trim and fill analysis. So, it is preferable to present both of the indicators to have a complete picture of the bias. |
| • All the method components of PRIMA checklist are not reflected in the method section your | Thanks for pointing this out. I can confirm that all points that needs to be pointed are clearly included and described in the revised manuscript. See the method section. |
| review. Please include all the components listed in the method section of the PRISMA.  • Please include data extraction your method section.  • Risk of bias or quality score for each study should be assessed by two reviewers. Please say something about this. Moreover. If there were any disagreement during quality assessment, how this was resolved it? If quality assessment was done by two reviewers, and if there were any disagreements, please do KAPA test? | Included in more clear way.  The quality of included studies was assessed by two independent reviewers and their score disagreement level was 1.5% (98.5% agreement level) and they reached to a consensus by discussion. It has been reported that both KAPA test and percent agreement have strength and limitations. However, by assuming that our disagreement level is very small and the risk of guessing among the reviewers is very low as they are well equipped in study appraisal, we preferred to report percent agreement level than KAPA and this approach has been well documented. See <https://www.ncbi.nlm.nih.gov/pmc/articles/PMC3900052/> |
| Results • The I2 value =96.7%, but in systematic review and meta-analysis, if the I2 > 75% it is better to report as a systematic review rather than merging heterogeneous results as a meta-analysis. How do you see this result?  • What is “the tooled” means?  • You tried to see the trend of depression over a time by dividing publication year. If you are interested to see the time trend in meta-analysis, I recommend you to do cumulative meta-analysis. Besides, to assess trend I recommend you to use study period rather than publication year.  • You included case control study to assess effect of antenatal depression on adverse birth outcomes. Can we assess effect using case control study?  • Discussion • Please remove sub-headings from discussion.  • “This might associate with the number of included studies during different trimesters, the pooled prevalence was high where large number of studies included and low where small studies were included as adjustment was not made on the number of studies”. This is the limitation of this review rather than your justification.  • “We found that antenatal depression prevalence increases from the first to the third trimester of pregnancy”. Any association between antenatal depression and physiological changes during pregnancy?  • “We noted that institutional based studies reported higher prevalence relative to community-based studies. So, how do you justify this result?  • Limitations • Only studies reported in the English language were included in your review. This is one limitation.  • What was your rational to exclude low quality papers?  • Conclusion • Be consistent and conclude according to your result. Besides, the conclusion part of your abstract is not in line with the conclusion part of in the main document.  • Finally, I recommend you to edit the paper again there are some spelling and grammar problems. | Thank you, editor. This is true indeed and we did not pool rather we based our discussion on the result from sub analysis in random effect model. It is also possible to identify the source of heterogeneity and conducting a sub-analysis. And we also believed that this approach is better to give further clue on the burden of the problem than just making our conclusion on descriptive results.  Sorry, this is editorial problem and this was to mean “Pooled”.  Thanks, this analysis approach was selected primarily for making the sub-analysis in more homogenies categories by using year of publication rather than having an objective of trend analysis. On the other side it might also gives you a clue about the trend of antenatal depression over time as the included year was used chronologically.  Thank you for raising this important issue. I hope so as case control study is intended to identify determinants of certain public health outcomes or problems. In this specific research topic, a researcher could identify determinants of low birth weight or preterm birth by interviewing postnatal mothers who had LBW or preterm birth as cases and those with normal births as a control and assessing a history of antenatal depression as a primary exposure variable. However, “effect” in this situation is not a causal effect rather just to imply association or relationship between antenatal depression and adverse birth outcomes. In this case we should have used association rather than effect which we amended in the revised manuscript as we included observational studies to see the association between these two public health problems.  Thanks. We used sub-headings as this review answered two big research questions that could separately reported and hoping that this use of sub-headings might improve the clarity of the discussion. However, to accept your recommendation we removed the headings and looking forward for your suggestion after having this in your mind.  Thanks. Yes, as you said not adjusting for the number of studies could be a limitation of the current review but on the other side as we did not intentionally review and included studies based on the time of screening what we most possibly do is summarizing what we found. So, different number of studies have been found conducted in different trimesters of pregnancy and these number of studies majorly affected the magnitude of pooled estimate across the categories leaving the comparison across time of pregnancy somehow in such limitation.  This part is not clear for me. However, to make it clear, studies found that due to the increase in number of hormonal and environmental risk factors during pregnancy, depression symptoms can also persist or the incidence might increase throughout the weeks of pregnancy.  This is the basic limitation of institutional based studies, they over estimate the true prevalence of health problems as individuals with the condition are more likely to visit a health facility as compared to healthy individuals. Similarly, in this case, pregnant mothers with different condition are more likely to visit a health facility and screening depression on such mothers might tend to overestimate the true prevalence of depression in the community. Sometimes, this could be explained in the opposite in mental health problems as this might also affect the health seeking behaviour of the mothers but in this context most of the mothers are considered as an opportunistic mother to be screened. Screening for depression in low- and middle-income countries is mostly conducted for research purposes as it is not so far considered as part of maternal services, so, mothers with maternal condition are more likely to come for follow up and as the same time they are more likely to experience stress or depression symptoms.  Thanks. Even though, trim and fill adjustment was used to minimize the overall risk of publication bias, we have included this concept as a limitation in the revised manuscript.  Thanks for raising this concern. This is a systematic review and meta-analysis and though it is possible to make a sub-analysis based on the quality of the included studies, we preferred to exclude them assuming that it is preferable to focus on quality evidences. As you might have an information, in the level of evidence, meta-analysis from high quality primary study is much better than a meta-analysis of any type of studies. So, we believe this review and meta-analysis have better level of evidence than the one that we could have done by including studies with low quality.  Thanks. I confirm that study conclusion was based on the main findings. Similarly, apart from the jungle of sentences included in the conclusion part of the body of the manuscript, similar points were there both in the conclusion of the abstract and the conclusion in the main document except the recommendation part that I have made revision in the revised manuscript. I am happy to revise if there are points apart from this if I get cleared about it.  Thanks for your keen observation.  Me and my supervisor went through the manuscript to address the spelling and grammar problems. |
